# Emotional Dysregulation in Anorexia Nervosa: Scoping Review of Psychological Treatments

**DOI:** 10.3390/healthcare12141388

**Published:** 2024-07-11

**Authors:** Enrica Cogodi, Jessica Ranieri, Alessandra Martelli, Dina Di Giacomo

**Affiliations:** 1 Postgraduate in Clinical Psychology, University of L’Aquila, 67100 L’Aquila, Italy; enrica.cogodi@graduate.univaq.it (E.C.); jessica.ranieri@univaq.it (J.R.); 2 Life, Health and Environmental Sciences Department, University of L’Aquila, 67100 L’Aquila, Italy; 3 Faculty of Biosciences and Agri-Food and Environmental Technologies, University of Teramo, 64100 Teramo, Italy; alessandra.martelli@unite.it

**Keywords:** emotion dysregulation, eating disorders, anorexia nervosa, psychological treatment, BMI

## Abstract

Eating disorders are complex psychiatric disorders characterized by compensatory and restrictive behavior and a preoccupation with one’s body. Eating and purging behaviors are considered dysfunctional emotional regulation strategies. Therefore, psychological treatment is essential. The most common psychological interventions are dialectical behavior therapy (DBT), cognitive–behavioral therapy (CBT), family therapy (FBT), multi-family group therapy (MFTG) and mentalization-based treatment (MBT). The aim of this study was to summarize the current evidence on the impact of psychological treatments on emotional regulation difficulties and psychological symptoms in patients with eating disorders, especially anorexia nervosa. A search was conducted on PubMed and Web of Science using the terms “anorexia nervosa” and “emotion dysregulation”. Of the 278 initial articles, we included 15 publications. The results indicate that the acquisition of coping strategies, through DBT, leads to an improvement in anxiety and alexithymia. DBT, CBT and MBT lead to a reduction in the use of dysfunctional emotional regulation strategies too. Eating disorders involve both physical and mental health; therefore, it is desirable for future research to focus on the mutual synergy between the mental and physical components by evaluating various factors, such as biomarkers and the most appropriate therapeutic approach, with respect to the treatment setting.

## 1. Introduction

Eating disorders (ED) are complex psychiatric disorders characterized by unhealthy exercise, a preoccupation with body weight, restrictive eating behaviors and compensatory behaviors. They are associated with compromised interpersonal functioning, poor quality of life, clinical and psychiatric comorbidity and high levels of mortality [1]. Usually, eating disorders occur at any age, but they are more frequent in early to mid-adolescence, especially among females [2].

According to the DSM-5 classification [3], the main EDs are anorexia nervosa (AN), bulimia nervosa (BN) and binge eating disorder (BED). BN is characterized by binge eating followed by purging behaviors such as vomiting or the use of laxatives and body concerns, which promote the maintenance of a normal body weight, while BED is characterized by recurrent episodes of binge eating (≥1/week for a minimum of 3 months) that promote the onset of obesity [4]. Meanwhile, AN is characterized by a refusal to maintain a healthy body weight, an intense fear of gaining weight, body image distortion and weight control behaviors such as excessive exercise and self-induced vomiting [5].

Regarding AN, two subtypes exist: the restricting subtype of anorexia (AN-R) and the binge–purge subtype of anorexia (AN-BP). The latter is characterized by binge eating or purging behaviors, such as self-induced vomiting or the use of laxatives, whereas the restricting subtype of anorexia nervosa is characterized by strict limitations in the amounts and types of food consumed [6].

According to Haynos and Fruzzetti’s model [7], some of the typical behaviors of patients with anorexia, such as binge eating, caloric restriction or purging, can be considered as maladaptive strategies to regulate aversive emotional states in social and emotional conflict. Therefore, AN can be described as a maladaptive emotion regulation strategy that attenuates psychological distress [8,9]. In fact, some authors have noted that patients with AN showed more negative emotions prior to and more positive emotions after restrictive eating, and exercise was associated with a decrease in negative affect after but not prior to physical activity. These findings indicate that a low-calorie diet and excessive exercise might be used as an emotional regulation strategy [8,10].

According to the multidimensional model proposed by Gratz and Roemer [11], emotional dysregulation is characterized by (a) difficulties in the awareness and understanding of emotions, (b) difficulties in the acceptance of emotions, (c) difficulties in controlling impulsive behavior and engaging in goal-directed behavior when experiencing negative emotions and (d) a lack of functional and appropriate emotional regulation strategies to modulate emotional responses in order to fulfil personal goals and situational demands.

Some evidence shows that individuals with anorexia nervosa have higher levels of alexithymia [12], deficits in emotional awareness and emotional clarity [13], difficulties in forming mental representations of emotions [14], problems in recognizing, expressing and understanding emotions [8], a lower ability to regulate emotions and fewer emotional regulation strategies to use when they are upset [15], more difficulties in regulating positive affective states [16], maladaptive beliefs about experiencing emotions that lead to the non-acceptance of emotional experiences [17], negative body self-image [1], difficulties in empathy [18] and interoceptive deficits [19].

In patients with ED, interoceptive deficits refer to difficulties in the identification of hunger signals and satiety and confusion between these body sensations and emotions [19]. Some common traits among individuals with AN, such as social isolation, cognitive rigidity, reward insensitivity, needs for symmetry and perfectionism, appear to be related to emotional overcontrol and they favor the maintenance of an eating psychopathology [12]. A lack of adaptive emotional regulation strategies, such as problem-solving skill difficulties, avoidance, suppression and rumination, was linked to a greater eating psychopathology [12].

Santos and Haynos [16] found that the greater non-acceptance of positive emotions was associated with greater anxiety and depression in AN and less restriction was linked to impulsivity in response to positive emotions. It has been hypothesized that anxiety could be associated with a fear of food and the subsequent avoidance and restriction of food in these patients.

Some studies have found no association between body mass index (BMI) and difficulties in emotional regulation for individuals with AN, while other studies have reported an association between emotional regulation deficits and underweight patients with AN compared to those in recovery. Other studies have found that BMI was positively associated with emotional regulation difficulties, the avoidance of affect and distress intolerance, and an improvement in emotional regulation deficits was associated with weight gain, independently of psychological treatment [20]. Further, it is hypothesized that patients with AN-BP have greater emotion regulation difficulties compared to patients with AN-R, but only in the emotional impulsivity area [21]. Other studies have investigated the differences between AN-R and AN-BP [1,8,12,16,21]. In AN-R, emotional regulation difficulties tend to be characterized by emotional overcontrol as a lack of emotion expression and emotional inhibition, while, in AN-BP, emotional regulation difficulties tend to be characterized by impulsivity and difficulties in inhibiting behavior when experiencing negative emotions [12].

Racine and Wildes [17] demonstrated that BMI was unrelated to emotional dysregulation, but depressive symptoms influenced changes in emotional regulation difficulties and AN symptoms. Further, depressive and alexithymic symptoms were associated with deficits in emotional regulation strategies in adolescents with anorexia, and alexithymia may be a negative prognostic factor for AN, independently of depressive symptoms and disease severity.

The role of emotional dysregulation in ED maintenance has received increased attention in both research and treatment. The most commonly investigated treatments in ED are dialectical behavior therapy (DBT), cognitive–behavioral therapy (CBT) and family-based treatment (FBT), followed by multifamily therapy group (MFTG) and mentalization-based treatment (MBT).

DBT is based on biosocial theory, according to which biological vulnerability and a poor environmental context cause difficulties in emotional regulation [22]. It aims to help individuals with AN to learn new coping strategies to effectively manage and regulate their emotions [23]. In CBT, the therapist focuses on alliance and motivation. Sessions aim to recognize and modify dysfunctional thoughts related to body image, weight and food. FBT focuses on family relationships and the ways in which parents can help their children to eat; furthermore, another aim of such treatment is to give the patient more responsibility for their own weight restoration [24]. MBT may enhance treatment strategies by adapting interventions to the mental capacities of a patient with ED and to help to maintain a therapeutic relationship and reduce relapse rates [25]. MFTG helps patients and their families to learn and reflect on one another’s thoughts, to identify and repair disconnections related to AN [26].

Despite an awareness that emotional dysregulation is a common phenomenon in eating disorders, the knowledge of the efficacy of psychological treatment in reducing emotional regulation deficits is currently poorly investigated. We therefore undertook our study to perform an evidence mapping review regarding the impact of psychological treatment on emotional dysregulation in eating disorder patients, particularly in patients with anorexia. Specifically, the research objectives of this review were to

- summarize the current evidence of the impact of psychological treatment on emotional regulation difficulties and psychological symptoms in eating disorder patients;
- identify gaps in the literature that may require further research;
- identify research questions in terms of the determinants for future implementation actions.

## 2. Materials and Methods

### 2.1. Study Design

We conducted an evidence mapping review to analyze the literature focused on emotional dysregulation associated with anorexia nervosa. The framework outlined by the Global Evidence Mapping (GEM) Initiative [27] in their methodological paper on scoping studies was adopted. We performed this review according to the Preferred Reporting Items for Systematic Reviews and Metanalysis (PRISMA)—Extension for Scoping Reviews (PRISMA-R) [28].

### 2.2. Search Strategy

To identify potentially relevant studies for inclusion, we performed a scoping search of MEDLINE through PubMed and Web of Science in March 2023. The key search terms were “anorexia nervosa” and “emotion dysregulation”.

### 2.3. Inclusion and Exclusion Criteria

To be considered eligible, the titles and abstracts of the retrieved studies had to refer to patients with anorexia nervosa and signs of emotional dysregulation. The range of the search strategy was restricted to the 2013–2023 period. To be included, studies had to (a) be published in the English language; (b) include psychological treatment; (c) include psychological measures. In addition, we excluded all reviews on the topic.

### 2.4. Article Selection and Data Extraction

To ensure the reliability of the review, the titles and abstracts of the retrieved studies were independently screened by two raters for eligibility. The same two raters also evaluated the full texts for inclusion that were retrieved by online databases and the faculty interlibrary service. Any disagreement between the raters was successfully resolved by discussion until a consensus was reached. The following data were extracted from each paper: (a) general information—authors, publication year, country and involved institution (e.g., university, medical center); (b) study characteristics—study design, sample size, target sample, range age; (c) biomarkers, comorbidity, psychological measures used and psychological treatment; (d) data related to the research question of the review—ED treatment program, a summary of outcomes.

### 2.5. Data Synthesis

The general information, the study characteristics and the psychological measures used and the data related to the research question of the review were descriptively synthesized. To explore the presence and the impact of emotional dysregulation in young adult patients with anorexia nervosa, the results were summarized referring to ED treatment program outcomes.

## 3. Procedure

### 3.1. Search Results

The literature search of the PubMed, Web of Science and Scopus electronic databases provided a total of 278 publications. After removing 44 duplicates, 234 references were identified for screening. Based on the inclusion/exclusion criteria, two reviewers screened all titles and abstracts for eligibility and successfully resolved disagreements by consensus. The full texts of the remaining 73 papers with potential for inclusion were examined comprehensively.

**Figure 1:**
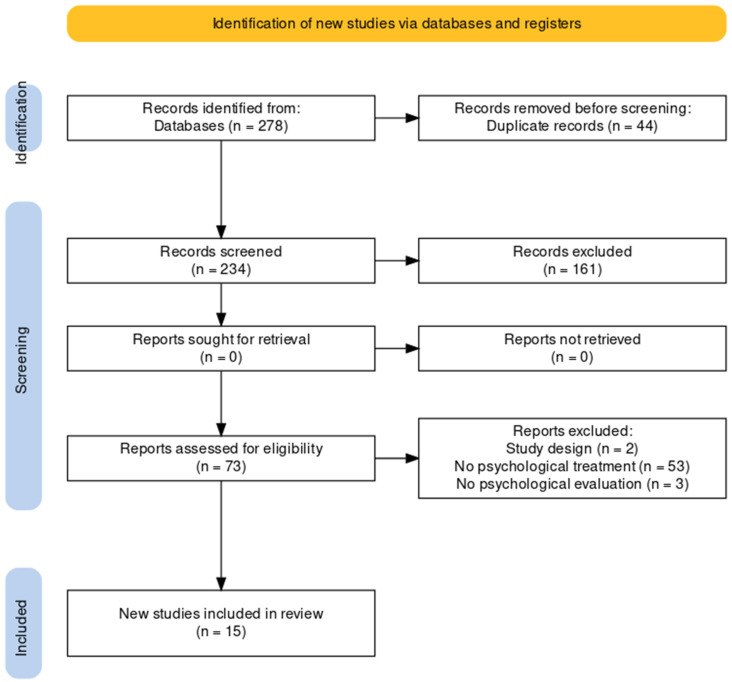
PRISMA flowchart of the study selection process for the review.

Out of a total of 73 articles assessed for eligibility, 58 were excluded and the reasons for exclusion were reported. Figure 1 shows all details of the study search and selection process. Finally, 15 studies were included.

### 3.2. Characteristics of the Included Studies

In Table 1, Table 2 and Table 3 the main characteristics of the included studies are summarized. We identified the following study characteristics: (a) study design, (b) authors, (c) eating disorders, (d) target sample, (e) sample size, (f) types of treatment, (g) duration of treatment and (h) number of dropouts.

All studies focused on patients with eating disorders and difficulties in emotion regulation. Regarding ED patients, the included studies involved patients with several conditions, including anorexia nervosa (AN), bulimia nervosa (BN), binge eating disorder (BED), eating disorders not otherwise specified (EDNOS), other specified feeding and eating disorders (OSFED), avoidant/restrictive food intake disorder (ARFID) and purging disorder (PD).

### 3.3. Sources of Articles

**Figure 2:**
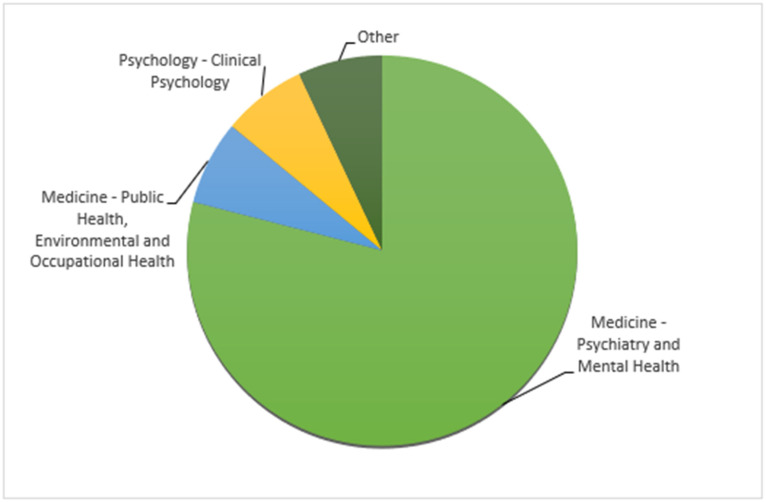
Subject areas of the included studies.

Figure 2 summarizes general information about the subject areas of the included studies. All papers were published in peer-reviewed journals from 2013 to 2023. Concerning the publication year, we noted a growing and emerging interest in the last ten years, showing a general intention to better understand the association between eating disorders and difficulties in emotional regulation; in fact, only one article, dated 2013, was found (6.7%), whereas five articles ware found dated 2022 (33%). We observed that almost all of the first authors’ institutions were universities (*n* = 13; 86%), highlighting the greater interest of academic staff rather than clinical staff.

## 4. Results

The 15 selected studies differed in their study design and methods and included research carried out using quantitative and qualitative designs.

Table 1, Table 2 and Table 3 report the details of the studies with, respectively, DBT, mixed treatment and other treatments. Table 4 and Table 5 report a summary of the included studies, respectively, with and without biomarkers. Table 6 reports the details of the measures and outcomes of the included studies. We describe the following features from each included paper: (a) authors; (b) biomarkers; (c) psychological measures; (d) comorbidities; (e) symptoms; (f) ED treatment program; and (g) outcomes. All papers focused on difficulties in emotional regulation and the use of strategies to regulate emotions in young adults with eating disorders, while the outcomes referred to psychological measures (e.g., anxiety, depression, emotional dysregulation, etc.) and the efficacy of the psychological treatment (e.g., dialectical behavior therapy, family-based therapy, cognitive–behavioral therapy, etc.).

As summarized in Table 4, the treatments mostly used in the studies with biomarkers were dialectical behavior therapy (DBT) and cognitive–behavioral therapy (CBT), followed by family-based treatment (FBT) and multifamily therapy interventions. Applying DBT, new coping skills such as mindfulness, distress tolerance, emotional regulation and interpersonal effectiveness were taught to help patients to regulate their emotions. The results showed a reduction in the use of dysfunctional emotional regulation strategies in AN and EDNOS patients, determined by the typical symptoms of the eating disorder. In fact, treatment was associated with an improvement in the adaptive expression of one’s emotions, a greater assumption of responsibility in decision-making and a reduction in self-harm and suicidal behavior [31]. The early acquisition of these skills (at one month) predicted a greater improvement in the ED pathology and depressive and emotional dysregulation symptoms at discharge [29]. Furthermore, the acquisition of cognitive and behavioral restructuring strategies produced a pre–post treatment improvement with respect to dissociative symptomatology, impulse regulation and body satisfaction in AN and BN patients [37].

Difficulties with emotion regulation were mostly treated during a mixed cognitive–behavioral, dialectical behavioral and interpersonal treatment in a sample of patients with AN who were admitted to a specialized intensive hospital-based treatment program. Patients with binge–purge anorexia nervosa (AN-BP), rather than the restricting subtype of anorexia nervosa (AN-R), reported greater difficulties with emotional regulation overall, particularly with refraining from impulsive behaviors when experiencing negative emotions. During the treatment, AN-BP patients achieved more pronounced improvements in impulse control compared to AN-R patients. With regard to emotional dysregulation, improvements produced by treatment significantly predicted changes in eating psychopathology over time, regardless of the impact due to increased body weight. The improvements found with respect to emotional clarity and involvement in goal-directed behavior in the presence of an altered emotional state corresponded to 36% of the improvements in eating psychopathology [12].

The behavioral intervention defined as Regulating Emotions and Changing Habits (REaCH) was focused on the creation of new behavioral routines and the elimination of maladaptive habits. This intervention was associated with a greater reduction in eating psychopathology and the severity of eating and physical activity habits, compared to supportive therapy alone, at the end of treatment in AN patients [32].

Neyman-Carlsson et al. [24] compared two types of psychological treatment: FBT and CBT. They showed that in the group of patients with anorexia who received family-based therapy (FBT), an impaired understanding of other people’s emotions predicted an increase in body weight; then, bulimic symptoms and emotional dysregulation were predictors of an increased number of diagnostic symptoms. However, in the group of patients undergoing CBT, a low level of emotional dysregulation and greater interoceptive deficits were able to predict changes in BMI.

The multifamily intervention called “Reconnecting for Recovery” (R4R) demonstrated its effectiveness in terms of improving eating psychopathology and emotion recognition in individuals with AN. These benefits were significant at the 6-month follow-up but not at the end of treatment. These findings demonstrate that changes in the ability to monitor, evaluate and modify emotional experiences take time [26].

Overall, AN, BN and BED patients undergoing DBT, FBT or CBT demonstrated an improvement in emotional reactivity compared to the control group [34] and a reduction in unhealthy physical exercise at two months from psychological treatment. Unhealthy exercise was prevalent among individuals with anorexia nervosa, as unhealthy exercise and restrictive eating may be used to avoid unpleasant and dysfunctional emotions. Increased emotional reactivity at two months was mediated by an improvement in emotion management, particularly in emotional avoidance, at one month after treatment. This effect was stronger for individuals with AN than other ED diagnoses [35]. Moreover, high emotional reactivity was associated with a greater reduction in overall eating symptoms and loss of control related to the severity of the symptomatology. However, emotional reactivity was not associated with binge eating or impairment-related changes in eating disorders [34].

Petersson et al. [33] compared the effectiveness of a psychological treatment called “affect school treatment”. Parallel to the intervention, the AS group and the control group received treatment as usual, which included individual psychotherapy (CBT). The “affect school treatment” included psychoeducation sessions on specific affective states like joy, fear, interest/startle, shame, anger, disgust and worry, followed by deep reflection. The last session focused on stress and pain education and their prevention. The AS group, rather than the CBT group, showed an improvement in alexithymic symptoms at the 6-month follow-up and a greater improvement in emotional regulation difficulties, eating-disorder-related thinking styles and behavior at the 6- and 12-month follow-ups.

AN patients undergoing mentalization-based therapy (MBT) showed an improvement in the general psychopathology of the eating disorder at discharge, both in terms of a reduction in bulimic symptoms and with respect to an improvement in BMI. Furthermore, the reduction in emotional regulation difficulties was evident both at discharge and at the 3-month follow-up. During the follow-up period, the reduction in general psychopathology was maintained, while there was a slight increase in eating pathology. Patients did not report an improvement in interpersonal function over the course of treatment but did so in the time period after discharge. The study found a strong association between the “Uncertainty” subscale of the Reflective Functioning Questionnaire (RFQ) and emotional dysregulation, bulimic symptoms and general psychopathology [25].

In the evaluation of the effectiveness of psychological treatment with respect to the emotional and psychological components assessed in the studies in which biomarkers were not recorded (Table 5), significant improvements in emotional regulation difficulties, anxiety, interoceptive deficits, binge eating and alexithymia emerged at the end of dialectical behavior therapy. Alexithymia manifested the cognitive symptoms of the eating disorder as rumination; therefore, an impairment in the ability to identify emotions appears to be correlated with the greater severity of the eating disorder [19,33].

Reilly et al. (2022) compared the effect of a combined intervention (psychological and pharmacological treatment) to that of psychological treatment alone [23]. The results showed that those who received both the psychological intervention and a mood stabilizer (lamotrigine) had a greater reduction in emotional and behavioral regulation difficulties than the control group, as well as in borderline personality disorder symptoms. Moreover, after lamotrigine administration, the patients showed the greater utilization of the skills acquired during dialectical behavior therapy.

Our review highlights the lack of attention in clinical practice given to clinical and psychosocial index measurement in the short and long term that are indicative of the eating disorder severity and quality of life. Moreover, future research could be oriented toward evaluating psychological treatments’ efficacy in psychoeducation about the impact of emotional regulation strategies on eating disorder management. Moreover, most of the studies focused on individual psychological treatment interventions’ efficacy, whereas few studies were conducted on group treatment interventions.

## 5. Discussion

In this review, we analyzed the effectiveness of psychological treatments in managing emotional dysregulation in patients affected by eating disorders. Scientific evidence shows that eating symptoms, restrictive and compensatory, can be defined as dysfunctional emotional regulation strategies. Therefore, psychological treatment is essential. 

CBT, DBT and FBT show appreciable levels of effectiveness in reducing eating symptoms associated with emotional difficulties; in particular, patients treated with DBT show increased skills for distress tolerance and more personal responsibility for life decisions [31]. The early acquisition of DBT skills in treatment predicted greater improvements in ED, depressive and emotional dysregulation symptoms [29]. Cognitive ED symptoms were associated with alexithymia symptoms, and this suggests that an impaired ability to identify emotions is related to more severe ED symptoms [36]. In reference to CBT, patients with lower levels of emotional dysregulation strategies, at the end of treatment, exhibited better outcomes [24]. In patients undergoing FBT, bulimic symptoms and difficulties with emotion regulation were predictors of increased diagnostic symptoms [24] and a reduction in alexithymic symptoms [36]. Moreover, for those who followed an MFTG, like R4R, it is highlighted that changes in emotional regulation strategies take time; in fact, the belief that one can access effective emotion regulation strategies was significant at the 6-month follow-up but not at the end of treatment. MBT was associated with an improvement in BMI, bulimic symptoms, interpersonal functioning and psychological quality of life [25]. For mixed treatment, it was found that when experiencing negative emotions, patients with the AN-BP subtype reported greater difficulties with impulse control and emotional regulation than those with the AN-R subtype. Moreover, the AN-BP subtype showed improvements in emotional clarity and engagement in goal-directed behaviors during the treatment, corresponding to improvements in eating disorder psychopathology [12]. Additionally, emotional reactivity was associated with a change in ED symptomatology and a loss of control related to eating severity during treatment, but not with ED-related impairments [34], and emotional avoidance at 1 month mediated the relation between unhealthy exercise at baseline and month 2 [35].

## 6. Conclusions

Applying a biopsychosocial approach [38] as a strategy to treat individuals who consider health (and illness) as a result of the complex and dynamic interaction of biological (genetic, biochemical, biological, organic), psychological (mood, personality, behavior) and social (cultural, family) factors, future research could develop intervention protocols and techniques that consider the combination of biomarkers and variations in relation to emotional dimensions; the integration of mental and physical health could promote improved well-being. The emerging needs in clinical practice are for a specific theoretical framework and the definition of a treatment setting tailored to the clinical and psychological characteristics of patients with eating disorders.

## Figures and Tables

**Table 1:** Main characteristics of the included studies with DBT.

Study Design	Authors	ED Patients	Target Sample	Sample Size	Type of Treatment	Duration of Treatment	N Dropouts
Observational study	Preyde et al., 2016 [19]	AN-R; AN-BP; BN; EDNOS	YO; AD	*n =* 104	DBT	Not specified	28
	Brown et al., 2019 [29]	AN-R; AN-BP; BN; BED; OSFED	YO; AD	*n =* 135	DBT	97.07 ± 53.16 days	65
	O’Mara et al., 2021 [30]	AN; BN; EDNOS	YO; AD	*n =* 54	DBT	11.3 ± 6.1 weeks	Not specified
Case Series Analysis	Federici et al., 2013 [31]	AN-P; AN-R; EDNOS	YO; AD	*n =* 7	DBT	6 months	Not specified
Quasi-experimental	Reilly et al., 2022 [23]	AN-BP; BN; OSFED	YO; AD	*n =* 62	DBT + pharmacological treatment	Not specified	1

Legend: AN-R = Anorexia Nervosa, Restricting Type; AN-BP = Anorexia Nervosa, Binge–Purging Type; BN = Bulimia Nervosa; EDNOS = Eating Disorder Not Otherwise Specified; BED = Binge Eating Disorder; OSFED = Other Specified Feeding and Eating Disorder; AN = Anorexia Nervosa; AN-P = Anorexia Nervosa, Purging Type; YO = Young; AD = Adult; DBT = Dialectical Behavior Therapy.

**Table 2:** Main characteristics of the included studies with mixed treatments.

Study Design	Authors	ED Patients	Target Sample	Sample Size	Type of Treatment	Duration of Treatment	N Dropouts
Pilot RCT/RCT	Steinglass et al., 2018 [32]	AN	YO; AD	*n =* 22	SPT; REaCH	1 months	1
	Neyman-Carlsson et al., 2019 [24]	AN	YO; AD	*n =* 78	CBT; FBT	18 months	4
	Petersson et al., 2022 [33]	AN; BN; BED; OSFED	YO; AD	*n =* 46	CBT; affect school	8 weeks	16
Observational study	Rowsell et al., 2016 [12]	AN	YO; AD	*n =* 108	CBT; DBT; interpersonal therapy	14.4 ± 7.1 weeks	52
	Bodell et al., 2022 [34]	AN; BN; BED; PD; ARFID	CH; YO; AD	*n =* 265	CBT; FBT	26 - 750 days	130
	Martin et al., 2022 [35]	AN; BN; BED; OSFED	CH; YO; AD	*n =* 127	FBT; CBT; DBT	Not specified	High rates
	Reilly et al., 2022 [36]	AN-R; AN-BP; BN; OSFED	CH; YO; AD	*n =* 1276	DBT; couples/FBT	83.27 ± 53.32 days	293
Case Series Report	Caslini et al., 2015 [37]	AN-R; BN; EDNOS	YO; AD	*n =* 8	Cognitive restructuring techniques; supportive psychotherapy	One year	Not specified

Legend: AN = Anorexia Nervosa; BN = Bulimia Nervosa; BED = Binge Eating Disorder; OSFED = Other Specified Feeding and Eating Disorder; PD = Purging Disorder; ARFID = Avoidant/Restrictive Food Intake Disorder; AN-R = Anorexia Nervosa, Restricting Type; AN-BP = Anorexia Nervosa, Binge–Purging Type; EDNOS = Eating Disorder Not Otherwise Specified; CH = Child; YO = Young; AD = Adult; SPT = Standard Supportive Psychotherapy; REaCH = Regulating Emotions and Changing Habits; CBT = Cognitive–Behavioral Therapy; FBT = Family-Based Treatment; DBT = Dialectical Behavior Therapy; Couples/FBT = Family-Based Treatment and Adjunctive Couples Therapy.

**Table 3:** Main characteristics of the included studies with other treatments.

Study Design	Authors	ED Patients	Target Sample	Sample Size	Type of Treatment	Duration of Treatment	N Dropouts
Pilot study	Tantillo et al., 2019 [26]	AN; OSFED	YO; AD	*n =* 10	MFTG	26 weeks	Not specified
Observational study	Zeeck et al., 2021 [25]	AN; BN; OSFED; CG	YO; AD	*n* = 38	MBT	2 years	5

Legend: AN = Anorexia Nervosa; OSFED = Other Specified Feeding and Eating Disorder; BN = Bulimia Nervosa; CG = Control Group; YO = Young; AD = Adult; MFTG = Multifamily Therapy Group; MBT = Mentalization-Based Treatment.

**Table 4:** Summary of included studies with biomarkers.

Authors	Bio Markers	Psychological Measures	Comorbidities	Symptoms
Federici et al., 2013 [31]	BMI; potassium; sodium; Qtc intervals	EDE-Q; DSHI	MDD; PTSD; ADHD; OCD; BPD; PD NOS; ANX NOS	ED symptoms; self-injurious behaviors
Caslini et al., 2015 [37]	BMI	EDI 2; DES; TAS-20	Obsessive Disorder; Histrionic Disorder; Borderline Disorder; Avoidant Disorder; Obsessive/Schizoid Disorder	ED symptoms; dissociative experiences; alexithymia
Rowsell et al., 2016 [12]	BMI	EDE-Q; DERS; BSI	Not Specified	ED psychopathology; emotional dysregulation; ANX; DEP
Steinglass et al., 2018 [32]	BMI	EDA-5; SCID-I; SRHI; EDE-Q; BDI; STAI-T; DERS; questionnaire to measure, at the beginning, the expectations of treatment and, at the end, treatment satisfaction post-treatment	Not Specified	DEP; ANX; ED severity; habit strength; emotional dysregulation
Brown et al., 2019 [29]	BMI	DBT-WCCL DSS; EDE-Q; BDI-II; DERS	Mood Disorder; Anxiety Disorder; Alcohol Use Disorder; Substance Use Disorder	DEP; severity of ED symptoms; emotional dysregulation; frequency of DBT skills
Neyman-Carlsson et al., 2019 [24]	BMI	EDI-3; RAB-R	Not Specified	ED symptoms; psychological traits
Tantillo et al., 2019 [26]	BMI	LEA; LAERS; MINI; EDE	Mood Disorder; Panic Disorder; Generalized Anxiety; PTSD	ED examination; acknowledge emotions; emotional regulation strategies
Zeeck et al., 2021 [25]	BMI	EDE; EDI-2; SCID-5; GSI; SCL-90-R; BFKE; IIP-K; DERS; CTQ; RFQ	Depression; Anxiety Disorder; OCD; PTSD; Somatoform Disorder; Dysthymia	Eating psychopathology; attachment; general psychopathology; interpersonal problems; emotional regulation; traumatization
Bodell et al., 2022 [34]	BMI	EDE-Q; LOCES-brief; CIA; DERS; ERS; CES-D	Not Specified	DEP; ED severity; loss of control; ED-related impairment; emotional regulation and reactivity
Martin et al., 2022 [35]	BMI	EDE-Q; AAQ-II	Not Specified	ED behaviors and cognitions; psychological inflexibility; experiential avoidance
Petersson et al., 2022 [33]	BMI	EDE-Q; DERS-36; TAS-20	Not Specified	ED cognitions and behaviors; emotional dysregulation; alexithymia

Legend: BMI = Body Mass Index; EDE-Q = Eating Disorder Examination Questionnaire; DSHI = Deliberate Self-Harm Inventory; MDD = Major Depressive Disorder; PTSD = Posttraumatic Stress Disorder; ADHD = Attention Deficit/Hyperactivity Disorder; OCD = Obsessive Compulsive Disorder; BPD = Borderline Personality Disorder; PD NOS = Personality Disorder Not Otherwise Specified; ANX NOS = Anxiety Disorder Not Otherwise Specified; ED Symptoms = Eating Disorder Symptoms; EDI-2 = Eating Disorders Inventory-2; DES = Dissociative Experiences Scale; TAS-20 = Toronto Alexithymia Scale-20; DERS = Difficulties in Emotion Regulation Scale; BSI = Brief Symptom Inventory; ANX = Anxiety; DEP = Depression; EDA-5 = Eating Disorder Assessment for DMS-5; SCID-I = Structured Clinical Interview for DMS-IV Axis I Disorders; SRHI = Self-Report Habit Index; BDI = Beck Depression Inventory; STAI-T = Spielberg State Trait Anxiety Inventory—Trait Version; DBT-WCCL DSS = DBT Ways of Coping Checklist—DBT Skills Subscale; DBT = Dialectical Behavior Therapy; EDI-3 = Eating Disorder Inventory-3; RAB-R = Rating of Anorexia and Bulimia Interview—Revised Version; LEA = Lack of Emotional Awareness; LAERS = Limited Access to Emotion Regulation Strategies; MINI = Mini-International Neuropsychiatric Interview; EDE = Eating Disorder Examination; SCID-5 = Structured Clinical Interview for DMS-5; GSI = Global Severity Index; SCL-90-R = Symptom Check-List; BFKE = Bielefelder Fragebogen zu Klientenerwartungen; IIP-K = Inventory of Interpersonal Problems; CTQ = Childhood Trauma Questionnaire; RFQ = Reflective Functioning Questionnaire; LOCES-Brief = Loss of Control Over Eating Scale-Brief; CIA = Clinical Impairment Assessment; ERS = Emotion Reactivity Scale; CES-D = Center for Epidemiologic Studies Depression Scale; AAQ-II = Acceptance and Action Questionnaire-II; DERS-36 = Difficulties in Emotion Regulation Scale-36.

**Table 5:** Summary of included studies without biomarkers.

Authors	Biomarkers	Psychological Measures	Comorbidities	Symptoms
Preyde et al., 2016 [19]	-	EDI-3; RAB-R	Not Specified	Emotional dysregulation; interoceptive deficits; ED risk
O’Mara et al., 2021 [30]	-	BAI; NMR	Not Specified	ANX; negative mood regulation expectancies
Reilly et al., 2022 [23]	-	BEST; UPPS-P; ERS; EDE-Q; WCCL; DBT skills use questionnaire	Major Depressive Disorder; Social Anxiety Disorder; PTSD	BPD symptoms; emotional reactivity; bulimic symptoms; negative urgency
Reilly et al., 2022 [36]	-	EDE-Q; TAS-20; BDI-II	Not Specified	DEP; alexithymia; ED cognitions and behaviors

Legend: EDI-3 = Eating Disorder Inventory-3; BAI = Beck Anxiety Inventory; NMR = Generalized Expectancy for Negative Mood Regulation Scale; BEST = Borderline Evaluation of Severity Over Time; UPPS-P = UPPS-P Negative Urgency Scale; ERS = Emotional Reactivity Scale; EDE-Q = Eating Disorders Examination—Questionnaire; WCCL = Ways of Coping Checklist; TAS-20 = Toronto Alexithymia Scale-20; BDI-II = Beck Depression Inventory-II; PTSD = Posttraumatic Stress Disorder; BPD = Borderline Personality Disorder; ANX = Anxiety; DEP = Depression; ED = Eating Disorder.

**Table 6:** Features of psychological treatment protocols in AN.

	Authors	Psychological Treatment	Program Target	Protocol Treatment	Statistical Analyses	Outcomes
Study with biomarkers and psychological treatment	Federici et al., 2013 [31]	MED-DBT	Emotional regulation	Group and individual sessions	No	Reduction in suicidal/self-injurious behavior, ED symptoms
	Caslini et al., 2015 [37]	Psychotherapy	Cognitive restructuring; emotional literacy and regulation	Weekly session	Wilcoxon test	Reduction in dissociative symptoms, impulse regulation and improved body satisfaction
	Rowsell et al., 2016 [12]	Cognitive behavioral interventions; interpersonal therapy; DBT skills training	Emotional regulation	Hybrid inpatient/day treatment	Independent samples *t*-tests; ANOVA; MANOVA; Mixed MANOVA	AN-BP improved more than AN-R in inhibiting impulsive behaviors
	Steinglass et al., 2018 [32]	REaCH	Cue awareness; emotional regulation	Twelve 45-min sessions three times per week	Pearson correlations; ANCOVA	REaCH was associated with reduction in eating disorder psychopathology and habit strength of dietary behaviors and physical activity
		SPT	psychoeducation; goal setting;			
	Brown et al., 2019 [29]	DBT	Mindfulness	5 h weekly group sessions	ANOVA; separate linear regression; RWA	Increased use of DBT skills; ED, depressive and emotional dysregulation symptoms
	Neyman-Carlsson et al., 2019 [24]	CBT	Identification and modification of patient’s dysfunctional thoughts and behaviors	60 1 h weekly sessions	*t*-tests; ANOVA; multiple and stepwise regression	In the CBT group, a lower degree of emotional dysregulation predicted a positive change in BMI. In the FBT group, an impaired ability to understand and respond to emotions predicted an increase in weight
		FBT	Developing functional family relations	Forty 90 min sessions		
	Tantillo et al., 2019 [26]	R4R	Development of emotional and relational processing skills	16 sessions of 90 min each	Within-patient standardized	R4R is an MFTG
	Zeeck et al., 2021 [25]	MBT	Reflective functioning; emotional regulation; interpersonal problems	Individual weekly session; one group session; a session with the family or partner	*t*-tests; multiple regression analysis; hierarchical stepwise regressions	Improvement in eating pathology, general psychopathology
	Bodell et al., 2022 [34]	CBT; FBT	Not detailed	Not detailed	ANCOVA; longitudinal multilevel models	Higher emotional reactivity was associated with loss of control, eating severity, ED severity and ED-related impairment
	Martin et al., 2022 [35]	CBT; FBT; DBT	Behavioral change	Not detailed	Pearson correlations; mediation model	Improvements in unhealthy exercise at month 2
	Petersson et al., 2022 [33]	Affect school treatment + CBT	Psychoeducation about a specific affect	Eight daytime 2 h group sessions	Pearson test; Spearman rank	The AS group reported a reduction in alexithymia, an improvement in eating disorder cognitions
Study without biomarkers and psychological treatment	Preyde et al., 2016 [19]	DBT	Emotional dysregulation	Not detailed	*t*-tests; bivariate linear regression	Improvement in the eating disorder and affective problems composite (APC)
	O’Mara et al., 2021 [30]	DBT	Mindfulness	Twice weekly sessions; group activities	Paired sample *t*-tests	Improvement in anxiety and a reduction in expectancies for negative mood dysregulation
	Reilly et al., 2022 [23]	DBT or DBT + lamotrigine	Emotional regulation	6 h a day for 5–6 days a week; skills group sessions	MLMs	Reduction in emotional reactivity, negative urgency and symptoms of borderline personality disorder
	Reilly et al., 2022 [36]	DBT	Not detailed	Individual therapy; biweekly skills groups	Two piece-wise multilevel models	Reduction in alexithymia
		FBT/couples therapy	Not detailed	Weekly sessions 10 h of treatment/day, 6 days a week		

Legend: MED-DBT = Multidiagnostic Eating Disorder Dialectical Behavior Therapy; ED = Eating Disorders; PTSD = Posttraumatic Stress Disorder; ANOVA = Analysis of Variance; MANOVA = Multivariate Analysis of Variance; AN-R = Anorexia Nervosa, Restricting Type; AN-BP = Anorexia Nervosa, Binge–Purging Type; DERS = Difficulties in Emotion Regulation Scale; REaCH = Regulating Emotions and Changing Habits; SPT = Supportive Psychotherapy; ANCOVA = Analysis of Covariance; DBT = Dialectical Behavior Therapy; RCI = Reliable Change Index; RWA = Relative Weights Analyses; ED = Eating Disorder; CBT = Cognitive–Behavioral Therapy; BMI = Body Mass Index; R4R = Reconnecting for Recovery; MFTG = Multifamily Therapy Group; AN = Anorexia Nervosa; MBT = Mentalization-Based Treatment; FBT = Family-Based Treatment; REML = Restricted Maximum Likelihood; IOP = Intensive Outpatient Program; PHP = Partial Hospitalization Treatment; MLMs: Multilevel Models.

## Data Availability

No new data were created or analyzed in this study. Data sharing is not applicable to this article.
